# Novel direct AMPK activator suppresses non-small cell lung cancer through inhibition of lipid metabolism

**DOI:** 10.18632/oncotarget.21716

**Published:** 2017-10-09

**Authors:** Xi Chen, Chun Xie, Xing-Xing Fan, Ze-Bo Jiang, Vincent Kam-Wai Wong, Jia-Hui Xu, Xiao-Jun Yao, Liang Liu, Elaine Lai-Han Leung

**Affiliations:** ^1^ State Key Laboratory of Quality Research in Chinese Medicine, Macau Institute for Applied Research in Medicine and Health, Macau University of Science and Technology, Taipa, Macau SAR, China; ^2^ Respiratory Medicine Department, Taihe Hospital, Hubei University of Medicine, Hubei, China; ^3^ Guangzhou Institute of Respiratory Disease, State Key Laboratory of Respiratory Disease, The 1st Affiliated Hospital of Guangzhou Medical College, Guangzhou, China

**Keywords:** novel AMPK activator, NSCLC, gefitinib-resistant, lipid metabolism

## Abstract

Drug resistance is becoming an obstacle in anti-cancer therapies. For target-based therapy of lung cancer, gefitinib, as the first generation of tyrosine kinase inhibitors (TKIs), demonstrated good initial response to the non-small cell lung cancer (NSCLC) patients whom harbors epidermal growth factor receptor (EGFR) mutation. However, within one year, additional EGFR mutation occurred, leading to eventual gefitinib-resistance. Therefore, it is urgently to discover novel effective small molecule inhibitors for those patients. Abnormal energy metabolism is accepted as new cancer hallmark. Recently, a metabolism rate-limiting enzyme 5’-adenosine menophosphate-activated protein kinase (AMPK) has become a promising anti-cancer target. In this study, we have identified a novel direct AMPK agonist, D561-0775 from a compound library by using molecular docking screening technique. We demonstrated that D561-0775 exhibited significant inhibitory effect on gefitinib-resistant NSCLC cell lines but less cytotoxicity on normal cells. Furthermore, D561-0775 demonstrated a remarkable *in vitro* AMPK enzyme activation effect. Taken together, D561-0775 showed potential anti-cancer activity via inducing apoptosis, cell cycle arrest, suppressing glycolysis and cholesterol synthesis after activation of AMPK in gefitinib-resistant H1975 cells. D561-0775 has provided a new chemical structure that could be developed as cancer drug for gefitinib-resistant NSCLC patients through inhibition lipid metabolism by directly targeting at AMPK directly.

## INTRODUCTION

Cancer is becoming a major life-threatening global public health problem [1], of which lung cancer as a particularly serious cancer form contributed to 20% of all cancer death cases [2, 3]. In addition, non-small cell lung cancer (NSCLC) is the most common histological type of lung cancer and dominates almost 85% of all lung cancer cases. Conventional therapy, such as surgery, chemotherapy, or radiotherapy, scarify normal cells during the curing progress are becoming unfavorable. To avoid harmful side-effect on normal cell, target-based therapy is the mainstream of modern cancer therapy. For example, gefitinib is a clinically-marketed drug which acts as tyrosine kinase inhibitor to inhibit epidermal growth factor receptor (EGFR) activating signaling pathway. For the NSCLC patients who harbor activating substitution from leucine to arginine at amino acid 858 (L858R) point mutation and in-frame exon 19 deletion on EGFR, response well to gefitinib and these two common mutation are used a biomarkers for gefitinib prescription. However, gefitinib-resistance commonly happens due to further substitution mutation from threonine to methionine at amino acid position 790 (T790M) occurred after one year or less gefitinib treatment. Therefore, developing new treatment strategy to tackle gefitinib-resistance is urgently.

To solve this, many researchers focused on inhibiting cancer progression by hampering energetic consumption status. Compared to normal cells, cancer cells prefer using glycolysis even in normal condition. This phenomenon was often referred as the “Warburg effect” [4]. As a major cellular fuel sensor, the 5’-adenosine menophosphate-activated protein kinase (AMPK) regulates metabolic homeostasis. It is a trimer enzyme made up of one catalytic subunit (α) and 2 regulatory subunits (β, γ). On activation, AMPK induces a series of metabolic changes to balance energy consumption and production intracellular. The essential role of AMPK has led to the development of numerous AMPK activators which might be used as novel drug candidates in the treatment of AMPK related disorders, diabetes, obesity, and other metabolic diseases, as well as cancer [5–9].

Activated AMPK adjusts its downstream channels through the cascade (e.g. acetyl-CoA carboxylase (ACC), mechanistic target of rapamycin (mTOR), tuberous sclerosis 1/2 (TSC1/2) to induce NSCLC cell death by producing material and energy situation [10]. AMPK could affect cell cycle [11], cell proliferation [12], and cell survival [10], also involve in the mitochondrial biogenesis [13] on NSCLC. AMPK responds to an increased AMP/ATP ratio by turning on ATP-generating pathway, while turning off ATP-consuming one [14]. It directly switches from an anabolic to a catabolic state to save emergency of lacking energy. Thus, AMPK could be activated by its upstream Ser/Thr protein kinase, mainly the tumor suppressor liver kinase B 1 (LKB1) and the calcium/calmodulin-dependent protein kinase kinase-β (CaMKKβ) [15, 16], low energy status (e.g. lack of oxygen, glucose or ATP) [14, 17, 18], and other AMPK activators. The mechanism of activation AMPK is ultimately to phosphorylate Threonine 172 site within the α-subunit [19]. It was reported lots of AMPK activators, which are divided into 2 types, direct and indirect AMPK activators, according to their different effective sites on AMPK structure. The indirect activators (e.g. 2-Deoxy-D-glucose (2DG), 5-Aminoimidazole-4-carboxamide ribonucleotide (AICAR) resveratrol, biguanides, curcumin, activate AMPK via activation of its upstream to achieve [20–23] and some of them are clinically in use. However, these activators are known to have additional molecular targets and AMPK-independent effects.

In some reports, indirectly agonist for example, biguanides anti-cancer function was showed as AMPK independent manner [24]. Latest, Vincent et al. compared 6 typical AMPK agonists including directly (salicylate, A-769662) and indirectly (metformin, phenformin, AICAR, 2DG) on cellular functions associated with proliferating cells. They established only synthetic activator A-769662 led apoptosis and inhibited proliferation through activation of AMPK. Other AMPK activators (salicylate, metformin, phenformin, AICAR, 2DG) of anticancer properties can not be attributed to AMPK dependent manner [25]. Those results implied that indirectly activate AMPK have unfavorable side effects. Thus, it is of highly importance to identify direct AMPK activator with effect on AMPK-dependent manner. Currently, very few direct activators were reported, for example, A-769662 and salicylate were reported to allosteric activate AMPK via straightly effecting AMPK's subunits [26, 27], however, they have not been launched into clinical trial yet at this moment.

In this study, we aim to apply high throughput molecular docking screening technique to identify novel AMPK direct activator from a compound database. Afterwards, we validated our compound by kinase-based and cell-based assays to confirm *in vitro* AMPK activation and anti-cancer effect of the compound on NSCLC cell lines. D561-0775, showed significantly direct activation of AMPK. In addition, it also exhibited anti-cancer activity on gefitinib resistant NSCLC cell line H1975, which provided a new compound for future anti-cancer therapy.

## RESULTS

### Alpha-AMPK activators are identified by molecular docking on a compound library

**Figure 1:**
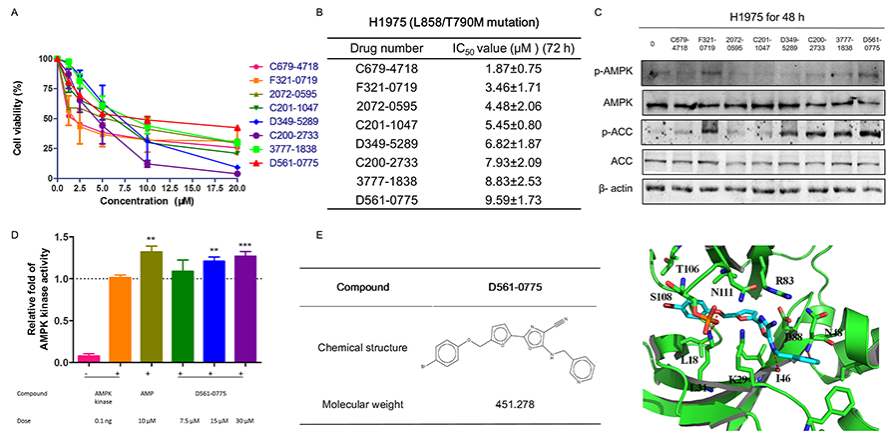
D561-0775 showed cytotoxicity on H1975, and had strongly activation effect on H1975 **(A)** The dose response curve of 8 compounds on H1975 cells after 72 h treatment. **(B)** IC_50_ values of 8 compounds on H1975 cells after 72 h treatment. **(C)** Western blot analysis of the protein level changes of p-AMPK, p-ACC, total AMPK, total ACC and β-actin after treatment with the 8 compounds for 48 h. **(D)** D561-0775 has a significantly activation of AMPK enzyme. **(E)** Chemical structure of D561-0775 and the binding mode of D561-0775 docked into AMPK. The AMPK protein was represented as cartoon. AMPK and key residues around the binding pocket were shown as sticks. The hydrogen bond was labeled as red dashed line. All data were expressed as mean ± SD (n= 3, ^**^ *p* < 0.01, ^***^ *p* < 0.001). All western blot images were cropped from full-length blots.

We have performed molecular docking analysis on 130,000 compounds library database, and chosen 74 compounds with high binding affiliation to AMPK kinase domain. Then 3-(4, 5-Dimethylthiazol-2-yl)-2, 5-diphenyl tetrazolium bromide (MTT) assay was performed to determine the growth inhibition rate of these 74 compounds on H1975 cells. All compounds have been analyzed by molecular docking, and tested on H1975 cells which harbor EGFR T790M/L858R double mutation that confers to gefitinib resistance. Preliminary screening was shown by treating H1975 with all the compounds at the concentration range from 0, 1.25, 2.5, 5, 10, and 20 μM for 72 h. Only 8 compounds showed IC_50_ values less than 10 μM and were shortlisted in ascending order in Figure 1A and 1B. We then further performed Western blot to examine whether these 8 compounds could activate AMPK by phosphorylating Threonine 172 site. The concentrations used for these 8 compounds were 2.5 μM, 5 μM, 5 μM, 5 μM, 10 μM, 10 μM, 10 μM, 10 μM, respectively, based on their IC_50_ value obtained from MTT assays. By comparison, D561-0775, showed the strongly AMPK activation efficacy among the 8 compounds (Figure 1C).

We further used the CycLex AMPK Kinase Assay Kit to measure the activation level of AMPK by D561-0775. Data showed that D561-0775 significantly activated AMPK, while AMPK active kinase and AMP were used as positive control. As shown in Figure 1D, the activity of D561-0775 was significantly higher as AMP at 30 μM *in vitro*, indicating that D561-0775 has AMPK activation activity.

The binding mode of D561-0775 docked into the active site of AMPK and chemical structure were shown in Figure 1E. The interactions between AMPK and D561-0775 mainly consist of hydrophobic, polar and hydrogen bond interactions. The hydrophobic groups of D561-0775 form hydrophobic interactions with the side chain of L18, L31, I46 and T106. The polar groups of D561-0775 form polar interactions with the side chain of I46, L31, N48, R83, D88, S108, and N11. In addition, D561-0775 also forms hydrogen bonds with the backbone of I46 and N48 and the side chain of L31. Molecular docking data showed that D561-0775 was a direct AMPK activator by binding to the kinase pocket.

### D561-0775 possesses potent cytotoxicity in gefitinib-resistant NSCLC cell lines

Since D561-0775 has the highest AMPK activation effect and showed cytotoxicity, we further tested in one more gefitinib-resistant NSCLC cell line (H820). H1975 contains L858R and T790M double mutations on EGFR. H820 harbors exon 19 in frame deletion and T790M double mutation on EGFR. These 2 cell lines are both insensitive to gefitinib. In order to know whether D561-0775 has cytotoxicity on EGFR wild type cell line, we have used H1299 EGFR wild type cells, and CCD-19Lu which is normal human lung fibroblast cell as control.

**Figure 2:**
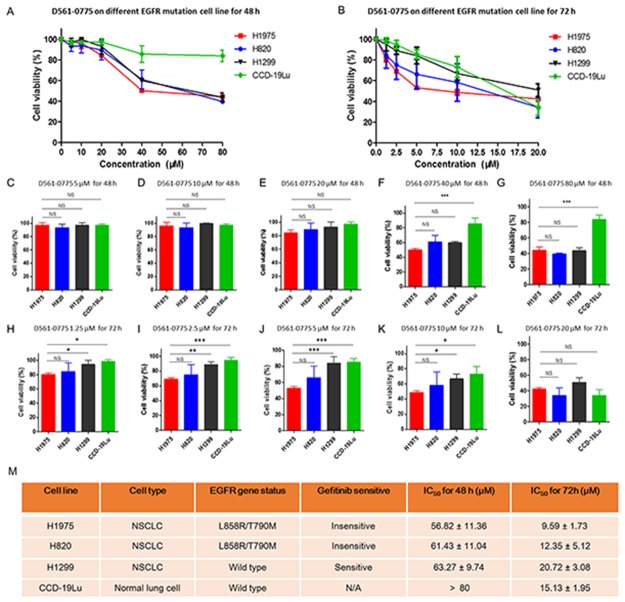
D561-0775 showed cytotoxicity on H1975, H820 and H1299 NSCLC cell lines, and normal lung cell CCD-19Lu **(A)** D561-0775 showed significant cytotoxic effect in a dose dependent manner on H1975, H820 and H1299, but did not affect CCD-19Lu at 80 μM after 48 h. **(B)** D561-0775 showed significant cytotoxic effect in a dose dependent manner on H1975, H820, H1299 and CCD-19Lu after 72 h treatment. **(C-G)** D561-0775 showed cytotoxicity in NSCLC cell lines at the concentration of 5 μM, 10 μM, 20 μM, 40μM, 80 μM after 48 h treatment. **(H-L)** D561-0775 showed cytotoxicity in NSCLC cell lines at the concentration of 1.25 μM, 2.5 μM, 5 μM, 10μM, 20 μM after 72 h treatment. **(M)** The IC_50_ values of D561-0775 on four cell lines were listed. All data was presented as mean ± SD (n= 3, ^*^ *p* < 0.05, ^**^ *p* < 0.01).

As shown in Figure 2A, after 48 h treatment, D561-0775 showed cytotoxicity on three NSCLC cell lines (H1975, H820 and H1299). The IC_50_ value of these three cell lines for 48 h treatment were 56.82 ± 11.36 μM, 61.43 ± 11.04 μM and 63.27 ± 9.74 μM. At 40 μM and 80 μM treatment dosages, it showed strong significantly difference in cytotoxicity between CCD-19Lu and H1975 cells (Figure 2C-2G).

After 72 h treatment, D561-0775 displayed cytotoxicity on all four cell lines. The IC_50_ value of these four cell lines were 9.59 ± 1.73 μM, 12.35 ± 5.12 μM, 20.72 ± 3.08 μM, 15.13 ± 1.95 μM, respectively for 72 h treatment (Figure 2B). Also, it showed significant difference when comparing EGFR mutant with EGFR wild-type cells (Figure 2H-2L). The EGFR status and IC_50_ value of each cell line after 48 h and 72 h treatment were presented in Figure 2M.

### D561-0775 activates the AMPK signaling pathway

**Figure 3:**
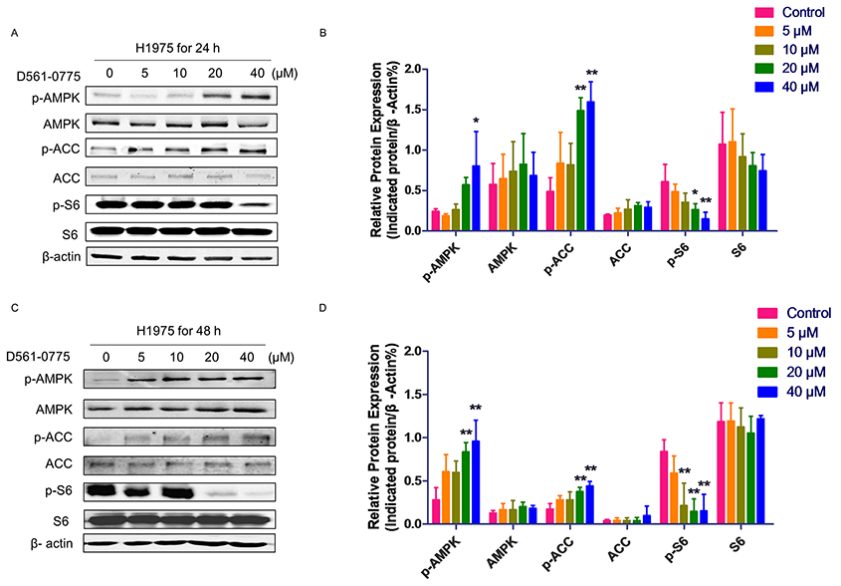
D561-0775 activated AMPK and its downstream signaling molecules **(A & C)** D561-0775 phosphorlated AMPK, ACC, and S6 on H1975 for 24 h and 48 h. **(B & D)** Statistical analysis of p-AMPK, AMPK, p-ACC, ACC, p-S6 after 24 h and 48 h. All data was presented as mean ± SD (n= 3, ^*^ *p* < 0.05, ^**^ *p* < 0.01). All western blot images were cropped from full-length blots.

To further prove if D561-0775 is an AMPK activator, the phosphorylation level of AMPK at Threonine 172 was detected by Western blot on H1975 cells after 24 h and 48 h treatment of the drug. Results showed that D561-0775 phosphorylated AMPK in a dose-dependent manner. Activation of AMPK signaling leads to inhibition of downstream mTOR pathway. Our result showed that p-S6, the downstream protein of mTOR was reduced. Also, as shown in Figure 3A and 3C, D561-0775 induced phosphorylation of another AMPK downstream substrate ACC which was widely used as an AMPK activation marker [14]. Statistical analysis by Figure 3B and 3D, densitometry of western blot data indicated a significantly increasing of p-AMPK, p-ACC, via decreasing p-S6 after treated by D561-0775 for 24 h and 48 h in H1975.

### D561-0775 effectively induces apoptosis in H1975 cells

**Figure 4:**
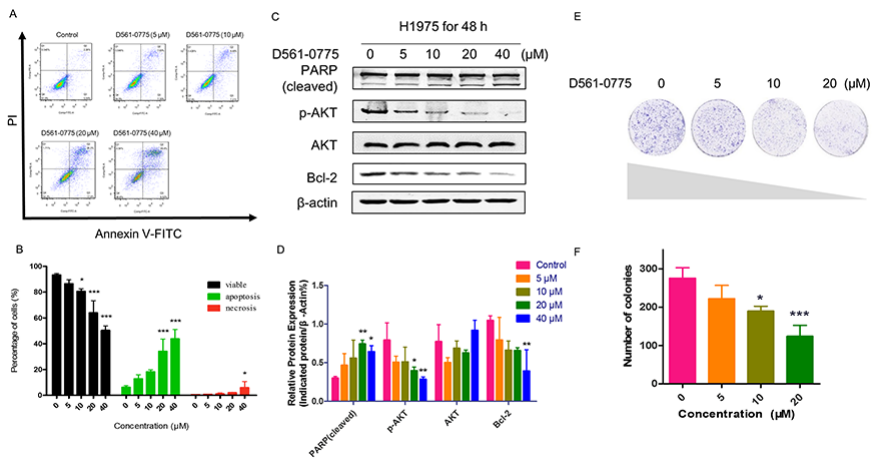
D561-0775 significantly induced apoptosis in H1975 **(A)** Flow cytometric analysis of the apoptosis level after D561-0775 treatment for 48 h. **(B)** Statistical analysis result of apoptosis data. **(C)** PARP was cleaved by D561-0775, while p-AKT, Bcl-2 were decreased by treating with D561-0775 for 48 h. **(D)** Statistical analysis of the densitometry of the signals of cleaved PARP, p-AKT, AKT, Bcl-2. **(E)** Photomicrographs of colony formation assay data after treatment with D561-0775 at different doses (0, 5, 10, 20 μM). **(F)** Statistical analysis of colony formation assay. All data was presented as mean ± SD (n= 3, ^*^ *p* < 0.05, ^**^ *p* < 0.01, ^***^ *p* < 0.001). All western blot images were cropped from full-length blots.

To investigate whether D561-0775 can induce apoptosis in H1975 cells, we measured the level of apoptosis in H1975 after D561-0775 treatment using flow cytometric analysis and western blotting assay. As shown in Figure 4, all of these results revealed that D561-0775 significantly induced apoptosis in H1975. In Figure 4A and 4B, from the flow cytometry results, there was only 7.81 % of cells undergoing apoptosis (Q2+Q3) in the control group, while cells were treated with 20 μM of D561-0775, apoptotic cells increased to 44.1% and in the 40 μM treatment group, 48.92 % cells were undergoing apoptosis. Moreover, as shown in Figure 4C and 4D, PARP was significantly cleaved and activated by D561-0775. While phopho-protein kinase B (p-AKT) were suppressed, and B-cell lymphoma 2 (Bcl-2) were decreased significantly, indicating induction of apoptosis.

Next, we further determine whether D561-0775 suppresses cell proliferation. Colony formation assay was performed. As shown in Figure 4E and 4F, D561-0775 treatment significantly attenuated colony formation in a dose-dependent manner. When cells were treated with 20 μM of D561-0775, the percentage of colony formed remarkably decreased 60 % when compared with the control group. It suggested that D561-0775 reduced cell proliferation and colony growth.

### D561-0775 causes cell cycle arrest in H1975 cells

**Figure 5:**
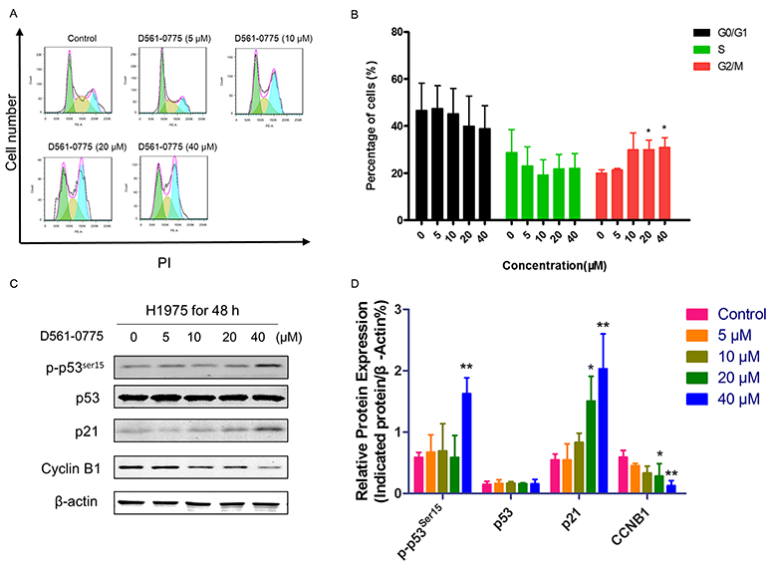
D561-0775 induced cell cycle arrest in H1975 cells **(A)** H1975 cells were treated with D561-0775 at different concentration for 48 h. **(B)** Statistical analysis of cell cycle distribution after 48 h drug treatment. **(C)** Western blot analysis of the protein levels of p-p53^ser15^, p53, p21, Cyclin B1 and *β*-actin after 48 h drug treatment. **(D)** Statistical analysis of the densitometry signals of p-p53^ser15^, p53, p21 and Cyclin B1. All data was presented as mean ± SD (n= 3, ^*^ *p* < 0.05, ^**^ *p* < 0.01). All western blot images were cropped from full-length blots.

To investigate the treatment mechanism of this AMPK agonist in H1975 cells, we further performed functional assays. To our knowledge, AMPK activation could awaken p53 [28], which has close relationship with p21. It arrested cell cycle during cell proliferation. After treatment with D561-0775 at the concentrations of 0, 5, 10, 20, 40 μM for 48 h, cells were harvested and stained with Propidium iodide (PI). Flow cytometry analysis showed that G2 phase arrested were induced at 20 μM and 40 μM respectively (Figure 5A and 5B). Then we detected the protein expression of cell cycle related proteins in H1975. From Figure 5C and 5D, p-p53^ser15^, p21 increased while Cyclin B1 (CCNB1) decreased. It suggested that D561-0775 induced G2 phase arrest by activation of the AMPK/p53/p21 pathway.

### D561-0775 suppresses glycolysis and cholesterol synthesis in H1975 cells

**Figure 6:**
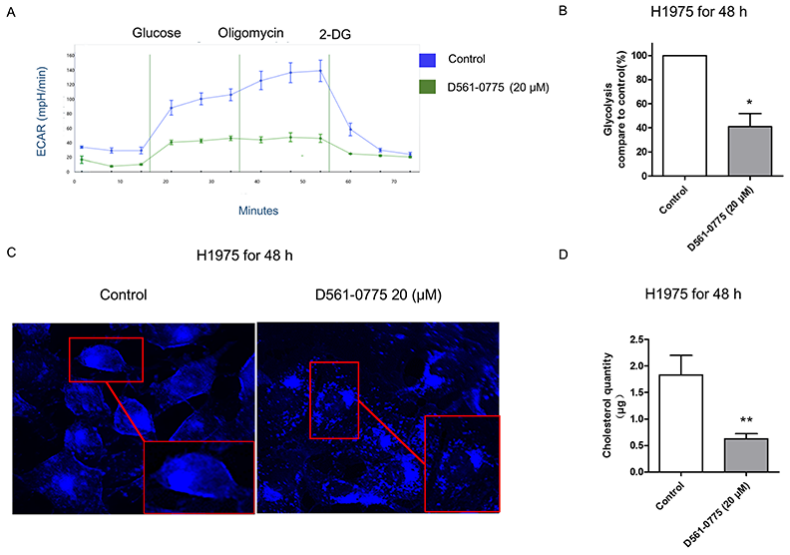
D561-0775 suppressed glycolysis and cholesterol synthesis in H1975 **(A)** The ECAR value decreased after treatment with D561-0775 for 48 h. **(B)** The statistical analysis result of glycolysis for 48 h. **(C)** Cholestrol staining after incubating with D561-0775 at 20 μM on H1975 for 48 h, magnified 40X dimension. **(D)** Quantification of the cholestrol level of H1975 after 48 h treatment with D561-0775. All data were presented as mean ± SD (n = 3, ^*^p < 0.05, ^**^ *p* < 0.01).

To test if D561-0775 could inhibit glycolysis, we treated H975 cells with D561-0775 at 20 μM for 48 h and compared the level of glycolysis with the untreated cells. We measured glycolysis by monitoring the extracellular acidification rate (ECAR) of cells in culture over 70 min. In Figure 6A and 6B, it showed that after treating D561-0775 for 48 h, the ECAR line and glycolysis ratio were lower than that of the control group. Since ACC inhibition was triggered by D561-0775, the fatty acid synthesis and cholesterol synthesis were likely to be suppressed. To probe this, the level of cholesterol was measured. As shown in Figure 6C, cholesterol was stained by filipin and presented as blue dots. Compare to the control group, the number of blue dots and the luminosity intensity decreased in the treatment group. Next, cholesterol quantity assay was presented to imply that after treated by D561-0775, the cholesterol quantity decreased in H1975 cells. Western blot showed that the level of fatty acid decreased after D561-0775 treatment (Figure 6D). All data were presented as mean ± SD (n = 3, ^*^p < 0.05).

## DISCUSSION

AMPK serves as a fuel sensor that plays an important role in regulating energy metabolism in all cells under physiological and pathological circumstances. Theoretically, activation of AMPK could help cells to pass the energy crisis [29]. In support of this, AMPK was found to be required to maintain cellular proliferation in astrocytic tumors [30], facilitate stem cell self-renewal by fomenting the glycolytic metabolism of pluripotent cells [31], and has been shown to be crucial for the maintenance of metabolic viability of myc-activated cells [32, 33]. Interestingly, after treating with a AMPK agonist, cancer cells were processing to death [34, 35]. Many recent studies have found that the cellular metabolic state alone, which is critically determined by AMPK activity, can dramatically affect multiple cellular processes relevant to carcinogenesis and cancer progression [36–38]. However, AMPK seems to be playing a dual role, either as a tumor promoter or a tumor suppressor, depending on the cellular context [39].

In this study, we have applied molecular docking and has identified a new direct AMPK activator, designated as D561-0775, which showed inhibition of Gefitinib-resistant NSCLC. By using AMPK enzyme activity assay, we confirmed that D561-0775 has remarkable AMPK activation ability. According to AMPK Kinase activity results, our data showed that p-AMPK increased by incubating with D561-0775 in H1975 gefitinib-resistant cells. After that p-ACC increased, while p-S6 as mTOR downstream was extraordinary decreased. ACC and mTOR were considered as a marker of activation of AMPK. They reflect different function in cell, ACC, one of three key enzymes is comprised in the de novo fatty acid biosynthesis pathway [40]. Many cancer cells exhibit a markedly increased rate of fatty acid synthesis that is specifically lower in normal cells except adipocytes. Activation of ACC inhibits fatty acid synthesis and promotes fatty acid oxidation. Fatty acid synthase (FASN) is a key biosynthetic enzyme involved in lipogenesis and the production of long-chain fatty acids from ACC. However, in rapidly proliferating cancer cells, fatty acids can be synthesized de novo in order to provide lipids for membrane formation and energy production via β-oxidation and lipid modification of proteins. Besides, the suppression of FASN expression by activation of AMPK was previously reported [41]. For example, AICAR reduced expression of FASN and ACC resulting in inhibition of proliferation on prostate cancer cells [42]. Combined with the increasing of p-ACC and cholesterol staining result, it revealed that D561-0715 inhibits fatty acid synthesis through activation of AMPK/ACC pathway. The mTOR is a serine/threonine protein kinase that regulates cell growth, cell proliferation, cell motility, cell survival, protein synthesis, autophagy, transcription [43]. Furthermore, overexpression of AMPK was also associated with a lack of mTOR level, resulting in suppression of cell growth, proliferation and protein synthesis [44–46].

To further examine how D561-0775 inhibited cell growth, we used quantitative flow cytometry analysis. It showed that D561-0775 remarkably increased the percentage of cells at G2 phase. That implied D561-0775 arrested cell cycle at G2 phase consequently inhibited mitosis. As a promising target, regulation of cell cycle checkpoints significantly contributed to treatment of cancer [47]. Whether cells can pass G2/M checkpoint, depends on the level of CCNB1, which is the initial activator and pivotal regulator, leading to regulation and activation of cyclin-dependent kinases 1 (CDK1)/CCNB1 complexes. Phosphorylation of these complexes further enforced cell cycle through the checkpoint and facilitated transition from G2 phase into mitosis [48, 49], which was consistent with our results. CCNB1 was decreased after treatment by D561-0775 48 h. In addition, activation of AMPK provides an inhibition effect on CDK and p21 [50]. It was reported that AMPK effected ataxia telangiectasia mutated (ATM) signals to mediate p53 and p21, targeted to cell cycle checkpoints [44, 51–54]. We observed that D561-0775 significantly increased p53 and p21, and it matches with our flow cytometry results of G2 phase cell cycle arrest. Taken together, our study indicated that D561-0775 induced H1975 cell arrest at G2 phase by AMPK activation to phosphate p53 and its downstream p21.

Consequently, apoptosis was observed after treatment with D561-0775, with suppression of AKT and Bcl-2, which belonged to the anti-apoptotic family [55]. Apoptosis played an important role in the development and homeostasis of multicellular organisms [56]. In the nervous system, apoptosis was required for normal development. AKT and Bcl-2 were considered anti-apoptotic factors. The relationship between AMPK and AKT was complex. It was reported that AKT could inhibit AMPK activation [57], while Cui's group found that AMPK inducing cell apoptosis by inhibit PI3K/AKT/mTOR pathway [58]. It has been reported that AMPK was a switch of p53, which was a tumor suppressor gene [59]. It now appears that the primary action of p53 in apoptosis is to directly and indirectly regulate the activity of the Bcl-2 family proteins [60]. These experiment results supported our results, which demonstrated D561-0775 could activate AMPK and downstream p53 leading H1975 cells to apoptosis. Thus, it may explain effect of the colony formation suppression by D561-0775.

In principle, the metabolic dependencies of cancer cells can be exploited for cancer treatment [61]. D561-0775 had cytotoxicity in gefitinib-resistant NSCLC cells. At 48 h, D561-0775 showed less toxicity on CCD-19Lu when comparing with EGFR mutant cells H1975 at 40 μM and 80 μM. D561-0775 has selectivity between NSCLC cells and normal cells at 48 h treatment time point. There is still significantly difference in cytotoxicity between the EGFR wild-type and EGFR mutant cells at low drug dosage. Treatment selectivity may be due to different glycolysis phenotype among these cells. Since, EGFR activation up-regulated glycolysis in EGFR mutant cells but not in EGFR wild-type cancer cells and normal cells, thus, it is predictable that AMPK activation will suppress cell viability more in EGFR mutant cells and normal cells. Thus, we further used glycolysis stress kit to examine the glycolysis inhibitory effect of D561-0775. Our result supported the view from Faubert et. al. They mentioned that AMPK activation could negatively regulated Warburg effect in tumor cells [62].

Although, some small molecules have been reported from the literature as direct AMPK activators (Table 1). According to these reports, there is no gefitinib-resistant cells used as research object. As A-769662 was confirmed as an AMPK activator on the β subunit [63], however, the catalytic site of AMPK locates at the α subunit [64]. Therefore, to design a compound directly binding to the α subunit can avoid the side effect. To our knowledge, there were only four α subunit binding activators being identified. Among these four, compound 991 was tested on AML cells only and was not tested on lung cancer, and the IC_50_ value of compound 991 is higher than D561-0775 [65]. Moreover, it was established that can not activate AMPK without the CBM domain, which is located at the β-subunit [63]. PT-1 was considered as the α subunit binding activator [66]. However it not only bind to the α subunit but also the γ subunit [67]. The effect of OSU-53 was only examined in thyroid carcinoma and myeloid-derived suppressor cells [68–71]. Compound 13 was shown to inhibit hepatic lipid synthesis via an AMPK-dependent manner [72], and inhibit Helicobacter pylori-induced oxidative stresses leading to gastric epithelial cell apoptosis [73] but has not been tested in cancer cell yet.

**Table 1:** Summary of reported direct AMPK activators

Compounds	Chemical structure	Binding subunit	Cell line	Effect time	Effect concentration	Reference
Salicylate	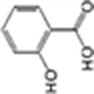	β	A549	48 h	103.64 ± 4.59 μM	[74]
A-769662	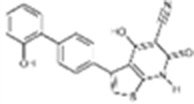	β	A549	48 h	10 μM	[13]
Compound 991	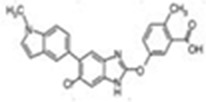	α β	AML	48 h	100 μM	[65] [63]
MT 63–78	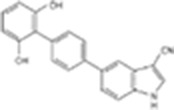	β	PC3	48 h	25-50 μM	[75]
PT-1	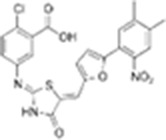	α γ	HelaHEK293	6 h1 h	20-40 μM100 μM	[66][67]
OSU-53	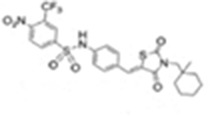	α	MDA-MB-231/MDA-MB-468	72 h24-72 h	5 μM5-10 μM	[71][69]
Compound-13	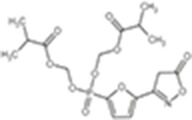	α	Mouse hepatocytesGastric epithelial cells	3 h24 h	10-100 μM10 μM	[72][73]
CNX-012-570	Not shown	β	HepG2/C2C12, 3T3L1	2-4 h	0.3 μM	[76]

Our compound is docking to bind on AMPK α subunit. Compare to these four activators, our compound D561-0775 chemical structure is simpler and could be easier to be synthesized. Small-molecule activators of AMPK will be invaluable for elucidating the functions of AMPK and validating the pharmaceutical importance of AMPK as a drug target. D561-0775 is a potential novel small-molecule activator that directly activates AMPK through regulation of α catalytic subunit and will be useful for evaluating the effects of AMPK under physiological and pathological conditions and studying its downstream signaling pathway.

In summary, our study has provided a new compound structure, D561-0775, which directly bind to AMPK α subunit and activate AMPK, exhibiting anti-cancer effect on gefitinib-resistant NSCLC cells. It induced apoptosis G2 phase arrest and suppressed glycolysis to inhibit cancer cell growth and proliferation. According our results, it suggested that D561-0775 is a potential and promising agent to treat gefitinib-resistant NSCLC patients by inhibition of glycolysis, cholesterol synthesis. Besides, it could be used potentially beneficial as tool drug for AMPK research.

## MATERIALS AND METHODS

### Materials

The compound library consisting 74 compounds were purchased from ChemDiv (San Diego, CA, USA). MTT powder and Dimethyl sulfoxide (DMSO) was purchased from Acros Organics (Morris Plains, NJ, USA). Adenosine 5′-Triphosphatase, filipin staining, chloroform and cholesterol quantity assay kit were purchased from Sigma Aldrich (St. Louis, MI, USA). Radioimmunoprecipitation (RIPA) lysis buffer (10 ×) and the primary antibodies of β-Actin, CCNB1, p-ACC, total ACC, p21, p-S6, total S6, p-p53^ser15^, PARP, p-AKT, total AKT and Bcl-2 were purchased from Cell Signaling Technology (Danvers, MA, USA). The primary antibodies of p-AMPK, total AMPK, were purchased from Santa Cruz (Dallas, TX, USA). The secondary antibodies of anti-rabbit and anti-mouse were purchased from Odyssey (Belfast, ME, USA). Fluorescein-conjugated goat anti-rabbit and mouse anti-bodies were purchased from Odyssey (Belfast, ME, USA) and Invitrogen (Waltham, MA, USA). Annexin V/ Propidium iodide (PI) staining kit was purchased from BD Biosciences (San Jose, CA, USA). RNase A was purchased from Sigma Aldrich (St. Louis, MI, USA). Ten percent fetal bovine serum (FBS), 100 U/ml penicillin and 100 μg/ml streptomycin were purchased from Gibco (Oklahoma, ME, USA). A complete mini, EDTA-free tablet was from Roche (Mennheim, Germany). DCTM protein assay kit was purchased from Bio-Rad (Hercules, CA, USA). Nitrocellulose (NC) membrane was purchased from GE Healthcare (Waukesha, WI, USA). Crystal violet was purchased from Amresco (Solon, OH, USA). Glycolysis stress kit was purchased from seahorse (Seahorse Bioscience, MA, USA). AMPK kinase assay kit and AMPK (α1/β1/γ1) active enzyme were from Cyclex. (CycLex Co., Ltd., Nagano, Japan).

### Cell culture

H1975, H820, H1299 NSCLC cell lines and CCD-19Lu normal lung fibroblast cell line were purchased from American Type Culture Collection (ATCC) (Manassas, VA, USA). The human normal lung fibroblast CCD-19Lu cells were grown in monolayer culture in MEM medium, whereas the three NSCLC cell lines were all grown in monolayer culture in RPMI 1640 medium. All culture media were supplemented with 10% FBS with 100 U/ml penicillin and 100 g/ml streptomycin. All cells were cultivated at 37 °C in a humidified atmosphere of 5 % CO_2_.

### Molecular docking analysis

Molecular docking calculation is performed to study the binding mode of D561-0775 to the adenosine 5’-monophsphate (AMP)-activated protein kinase (AMPK) using Glide program in Schrodinger software (Schrodinger, Inc., New York, NY, 2009). The structure of D561-0775 was processed by the *LigPrep* based on OPLS-2005 force field. The 3D structure of AMPK for molecular docking was retrieved from the Protein Data Bank (PDB ID: 4CFE) and was processed by the *Protein Preparation Wizard* module. D561-0775 was docked into the binding site of the AMPK with the standard precision (SP) scoring mode. The docking grid box was defined by centering on the compound 991 in the complex of AMPK and ligand (PDB ID: 4CFE). We used compound 991 as positive control which binds to α subunit.

### MTT assay

Cells were seeded with 4 × 10^3^ and 3 × 10^3^ cells/well in a 96-well plate and allowed to adhere overnight, respectively. Cells were treated with various concentrations of compounds (0, 5, 10, 20, 40, 80 μM) with DMSO as vehicle control for 48 h and (0, 1.25, 2.5, 5, 10, 20 μM) for 72 h. Ten μl of MTT solution were added to each well and incubated at 37 °C for 4 h. Then, 100 μl of resolved solution (10 % SDS and 0.1 mM HCL) was added to each well and incubated at 37 °C for 4 h to solubilize the formazan crystals. Absorbance of plates was measured at 570 nm (absorbance) and 650 nm (reference) with Tecan microplate reader (Morrisville, NC, USA).

### AMPK enzyme activity assay

We used the CycLex AMPK Kinase Assay Kit to detect the AMPK activation activity of D561-0775. According to the manufacturing instructions, on a 96-well plate, 0.2 ng of AMPK (α1/β1/γ1) active enzyme was added into each well with D561-0775 (7.5, 15 & 30 μM) or 10 × of positive control, AMP (100 μM) in kinase assay buffer (50 μM ATP & 10 mM DTT) and were incubated at 30°C for 20 min. The reaction was then stopped by washing with buffer for 5 times. Then, anti-phospho-mouse IRS-1 S789 monoclonal antibody was added to each well at room temperature for 30 min. After washing with buffer for 5 times, HRP-conjugated anti-mouse IgG was added to each well at room temperature for 30 min. After washing with wash buffer, the substrate reagent was incubated in wells at room temperature for 5-15 min. Stop solution was added to each well before measuring absorbance at 450/550 nm.

### Western blot analysis

H1975 cells were plated at a cell density of 1.5 × 10^5^ cells/well at a 6-well plate and cultured overnight for attachment. Cells were harvested after stimulated by D561-0775 in different dosage for 48 h. Cells were lysed in 1 × RIPA lysis buffer with proteinase inhibitor and phosphatase inhibitors added, and were scraped off from the plate by a plastic cell scraper. All the lysate was mixed well and kept in ice then transferred to a new tube. The lysate was centrifuged at 4 °C at 12000 rpm for 5 min. After centrifugation, the suspension with protein lysate was kept on ice. Protein concentration was quantitatively measured by DCTM protein assay kit, the supernatant was transferred into a new tube and mixed with 5 × loading buffer. Each sample was boiled at 100 °C for 5 min. Twenty-five μg of each protein samples were loaded into the well of a 10 % SDS-PAGE gel with one lane of 3 μl protein molecular weight marker. The gel was run for 20 min at 80 V for stacking, and then added to 120 V for protein separation. After separation, the proteins from the gel were transferred to a NC membrane for 2 h at 300 mA, the membrane was blocked with 5 % non-fat milk diluted with 1 × TBST (0.1 % Tween 20 in Tris-buffered saline) at room temperature for 1 h and washed with 1 × TBST for three times. Membranes were incubated with primary antibodies at 1:1000 dilution at 4 °C overnight. After washing the membrane 3 times with 1 × TBST, the membranes were incubated with secondary antibodies at 1:10000 dilutions for 1 h at room temperature. GAPDH was used as endogenous loading control for normalization. The signal intensity of the membranes was detected by LI-COR Odyssey scanner (Belfast, ME, USA).

### Cell cycle analysis

H1975 cells were plated with 1.5 × 10^5^ cells/well at a 6-well plate and cultured overnight for attachment. Cells were treated with D561-0775 at 0, 5, 10, 20, 40 μM for 48 h. After treatment, all cells were harvested by trypsinization, and collected by centrifugation. After removing all suspension, cells were washed by PBS. Cells pellets were re-suspended in 70 % ethanol at 4 °C overnight. Cells were centrifuged at 1000 rpm for 5 min to remove all the ethanol. Each cell pellet was re-suspended in 500 μl PI staining solution at 37 °C for 30 min in dark. Then, they were washed in PBS twice. Cells were re-suspended in 300 μl 1 × binding buffer and transferred to the flow cytometer (BD FACS Aria III).

### Apoptosis assay

H1975 cells (1 × 10^5^ cells /well) were seeded in a 6-well plate for 24 h, and treated with the indicated concentrations of D561-0775 for an additional 48 hours at 37°C. After 48 h, the cells were washed by ice-cold 1 × PBS once and harvested by trypsinization. Then cells were centrifuged, collected and resuspended in ice-cold 1 × PBS. After removing the supernatants, cell pellets were re-suspended in 100 μl 1× Annexin -binding buffer. The cells were then double-stained with Annexin-V FITC and PI (100 μg/mL) of 2 μl respectively for 15 min at room temperature in dark. After that, 300 μl 1× Annexin-binding buffer was added to re-suspension. Apoptotic cells were quantitatively counted by a BD Aria III Flow Cytometer (BD Biosciences, San Jose, California, USA).

### Glycolysis stress test

Glycolytic flux analysis was performed using the XF Glycolysis Stress Kit with the Seahorse Biosciences XF analyzer (Seahorse Bioscience, MA, USA). H1975 cells were trypsinized and 3×10^3^ cells were plated per well of assay mini plate. After 48 h of D561-0775 treatment, medium was replaced with assay medium and incubated at 37°C without CO_2_ for 1 h to equilibrate the assay. After equilibration, three well-defined small molecule modulators of glycolysis, glucose and oligomycin (both are promoters of glycolysis) as well as 2-Deoxy-D-glucose (2DG) (a glycolysis inhibitor) were sequentially administered. Glycolysis leads to proton extrusion from the cells which results in the acidification of surrounding media, so the more glycolysis increasing, the pH range changed more strongly. The ECAR-extra cellular acidification rate was measured at specific time points and profile of the glycolytic flux were recorded in real time.

### Cholesterol staining assay

H1975 cells were grown on glass coverslips in 6-well plate, each well of 1 × 10^5^ cells /well. After treatment with D561-0775 at 20 μM for 48 h at 37°C. Cells were washed 3 times with PBS and then fixed with 4 % paraformaldehyde (PFA) in PBS for 1 h. Cells were rinsed 3 times with PBS and incubated with 1 ml of 1.5 mg glycine/ml PBS for 10 min at room temperature. Cells were stained with 1 ml of filipin working solution for 2 h at room temperature in dark, then were washed with PBS for 3 times. Coverslips were mounted onto microscope slides with fluor save regent. Images were captured with Delta Vision Live Cell Imaging System with 40× objective magnification.

### Cholesterol quantity assay

The quantification of cholesterol were determined using Sigma kit. Briefly, cells were harvested in chloroform buffer. The samples were centrifuged at 13000 g for 10 min to remove insoluble debris. Organic was collected and allowed to air dry on 50°C for 30 min. Samples were vacuum dried for 2 h to remove traces of chloroform. The dried lipids were resuspended via vortexing in fatty acid assay buffer. Added 50 μl sample to 96 well plate. Then added testing buffer into sample, mixed for 60 min without light in 37°C. Samples further quantified by measuring absorbance at 670 nm.

### Colony formation assay

H1975 cells were seeded at a density of 1 × 10^3^ cells/well in a 6-well plate, and cultured overnight for attachment. Cells were exposed on D561-0775 with concentration (0, 5, 10, 20 μM) for 10 days. Then medium was changed at every 72 h. At the 10^th^ day, the colonies were washed by PBS once, and fixed with 4 % PFA. The colonies were washed by PBS twice, stained with crystal violet solution for 20 min, and washed. The number of colonies formed were counted in each group.

### Statistical analysis

All data represent mean values of at least three independent experiments and were expressed as mean ± SD. The statistical significant differences were analyzed by one-way ANOVA followed by Dunnett's test for comparison tests, using Graph Prism Version 6.0 software (San Diego, CA, USA). ^*^ *p* < 0.05, ^**^ *p* < 0.01, ^***^ *p* < 0.001 were considered as significant.

## Abbreviations

ACC: acetyl-CoA carboxylase
AICAR: 5-Aminoimidazole-4-carboxamide ribonucleotide
AKT: protein kinase B
AMPK: 5’-adenosine menophosphate –activated protein kinase
Bcl-2: b-cell lymphoma 2
CaMKKβ: calcium/calmodulin-dependent protein kinase kinase-β
CCNB1: cyclin-dependent kinase 1
ECAR: extracellular acidification rate
EGFR: epidermal growth factor receptor
FASN: fatty acid synthase
LKB1: liver kinase B 1
mTOR: mechanistic target of rapamycin
NSCLC: non-small cell lung cancer
PARP: poly ADP ribose polymerase
TSC1/2: tuberous sclerosis 1/2
2DG: 2-Deoxy-D-glucose
